# Comparison of complication types in patients receiving vesicant intravenous antimicrobials or vasopressors via midlines and peripherally inserted central catheters

**DOI:** 10.1017/ash.2024.363

**Published:** 2024-09-05

**Authors:** Bryan Grigg, Nishant Varghese, Christi Knapp, Sabra L. Shay, Geraldine Jones, James P. Herlihy, Prasad Manian, Bradley Lembcke, Mayar Al Mohajer

**Affiliations:** 1 Baylor College of Medicine, School of Medicine, Houston, TX, USA; 2 Baylor St. Luke’s Medical Center, Houston, TX, USA; 3 Department of Clinical Intelligence, Premier Inc., Charlotte, NC, USA; 4 Department of Medicine, Section of Pulmonary and Critical Care, Baylor College of Medicine, Houston, TX, USA; 5 Department of Medicine, Section of Infectious Diseases, Baylor College of Medicine, Houston, TX, USA

## Abstract

We assessed adverse events in hospitalized patients receiving selected vesicant antibiotics or vasopressors administered through midline catheters or peripherally inserted central catheters (PICC). The rates of catheter-related bloodstream infections, thrombosis, and overall events were similar across the two groups, while occlusion was higher in the PICC group.

## Introduction

Peripherally inserted central catheters (PICC) are frequently used to obtain durable intravenous access due to their ease of insertion, relative safety, and facilitation of outpatient parenteral antimicrobial therapy. However, in hospitalized patients, PICC have a similar risk of catheter-related bloodstream infections (CRBSI) as central venous catheters (CVC).^1,2^ Midline catheters (MC) have emerged as a safer alternative and are associated with reduced rates of CRBSI compared to PICC but may convey an increased risk of thrombosis.^3,4^

A key component when choosing line type is the drug to be administered. Until the last decade, vesicant drugs such as vasopressors and antibiotics have been limited to administration via central catheters. Consequently, the effect of vesicant drugs on adverse events in MC is unknown.^5–9^ Thus, we aimed to compare rates of CRBSI, thrombosis, occlusion, and overall adverse events in hospitalized adult patients receiving select antibiotics and/or vasopressors through PICC or MC.

## Methods

This retrospective chart review comprised patients admitted to intensive care units (ICU) at a quaternary medical center in Texas between April 2021 and September 2022. We included patients who received ciprofloxacin, levofloxacin, meropenem, vancomycin, dobutamine, dopamine, epinephrine, milrinone, norepinephrine, phenylephrine, and/or vasopressin through either a PICC or MC. Based on our hospital policy, these medications were allowed to be administered in either PICC or MC. Patients who received CVC or both PICC and MC during their stay were excluded.

We evaluated all positive blood cultures collected between 2 days after line insertion and up to 2 days after line removal. Two authors (BG and MA) reviewed charts to adjudicate whether the positive blood cultures were secondary to line infection (CRBSI), blood culture contamination, or an alternative infection source (eg, intraabdominal infection or pneumonia). Thrombosis was defined as either symptomatic deep vein thrombosis (DVT) or superficial venous thrombosis (SVT) in the ipsilateral arm to the catheter diagnosed 2 days after line insertion and up to 2 days after line removal. Occlusion was defined as the need for intra-catheter administration of alteplase (2 mg) for which an alternative indication was not documented. Device days were defined as days in which a line was in place and were not necessarily continuous. Days in which patients had multiple devices of one type inserted were counted as a single day.

The study outcomes included CRBSI, thrombosis (DVT or SVT), occlusion, and overall adverse events. Incidence rate comparison, 30-day Kaplan-Meier survival curves, and the log-rank test were used to compare incidence rate ratios (IRR) or hazard ratios (HR), as appropriate, between patients receiving MC and PICC. Day 0 was considered the date of line insertion, and patients who died within 30 days were censored. Multiple Cox proportional hazard regression models were employed to adjust for potential confounding factors, including age, gender, ICU type (medical vs surgical), and duration of antibiotics and vasopressors. An alpha level of 0.05 was used to determine statistical significance. The analysis was done using R version 4.2.2 (Vienna, Austria).

## Results

A total of 445 patients were included, with 359 (80.7%) receiving select antibiotics and/or vasopressors via MC and 86 (19.3%) via PICC. There were 3,910 device days in the MC group, versus 987 in the PICC group. All lines had documented insertion and removal dates. The overall median device days were 9 (interquartile range [IQR] 5–14), with MC and PICC in place for a median of 8 (IQR 5–14) and 9 days (IQR 6–14), respectively. Patient characteristics were similar across the two device types in age, gender, race/ethnicity, and unit (medical vs surgical ICU). The median antibiotic and vasopressor days of therapy (DOT) were 3 (IQR 1–5) and 0 days (IQR 0–2) in the MC group compared to 3 (IQR 1–6; *P* = 0.549) and 0 (IQR 0–2; *P* = 0.1) in the PICC group, respectively.

The incident rate for CRBSI was 0.18 infections per device day (0.2 in the MC vs 0.1 in the PICC; IRR 2.02; 95% CI, 0.27, 89.61). The incident rate for thrombosis was 0.43 (0.49 in the MC vs 0.2 in the PICC; IRR 2.40; 95% CI, 0.58, 21.23). Regarding occlusion, the incidence rate was 0.29 (0.1 in the MC vs 1.01 in the PICC; IRR 0.1; 95% CI, 0.02, 0.35).

**Figure 1.**
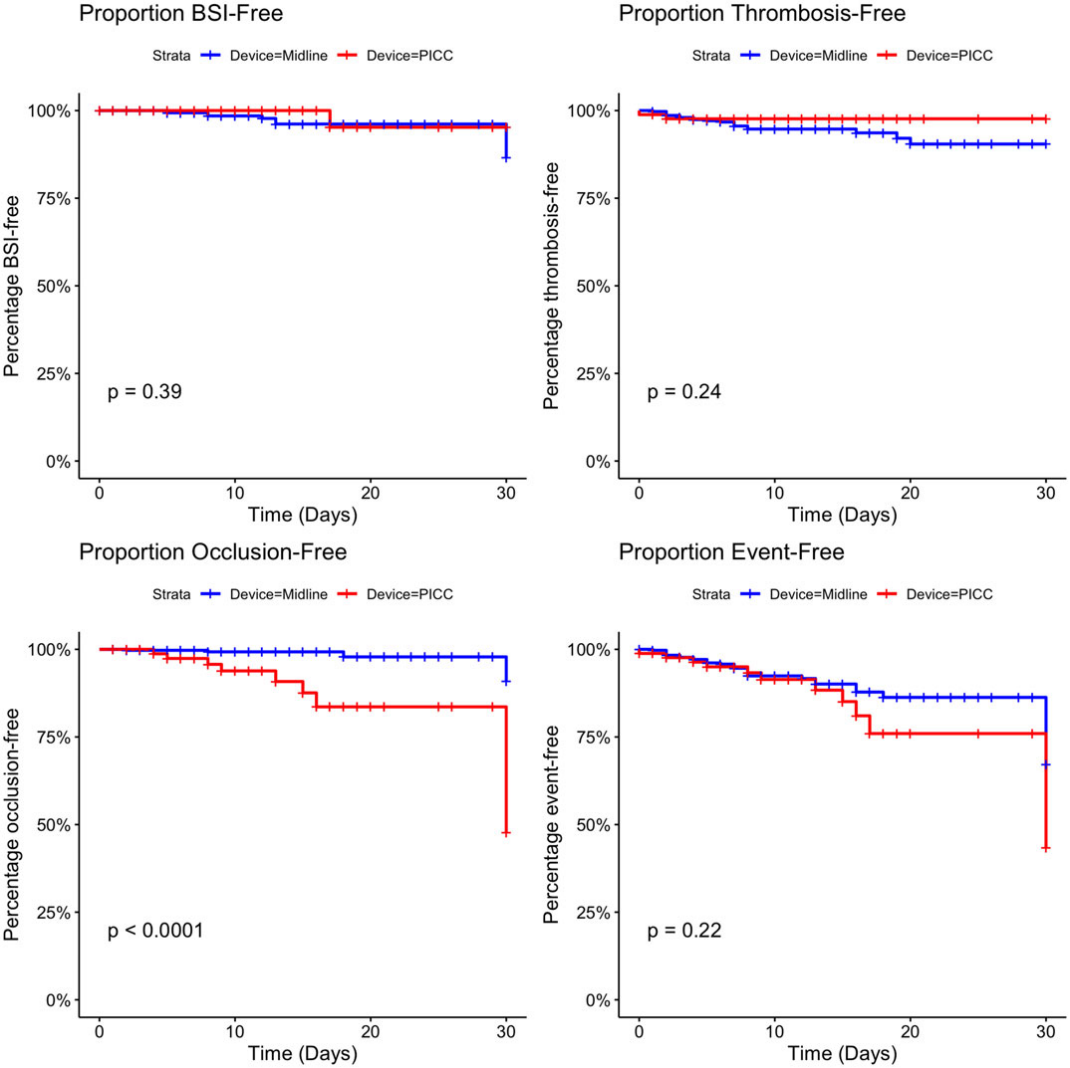
Kaplan-Meier survival curves comparing MC and PICC. This figure represents the survival rates for the 2 study groups: midline catheters (blue) and peripherally inserted central catheters (red). Four outcomes are shown: bloodstream infections (top left), thrombosis (DVT or SVT) (top right), occlusion (bottom left), and all events (bottom right). The log-rank *P* values are presented in each panel. MC, midline catheters; PICC, peripherally inserted central catheters; BSI, bloodstream infections; DVT, deep vein thrombosis; SVT, superficial vein thrombosis.

There were no differences in HR of CRBSI (Figure 1, *P* = 0.39), thrombosis (*P* = 0.24), or total events (*P* = 0.22) across the 2 study groups; however, the HR for occlusion was higher in the PICC compared with the MC (*P* < 0.001). The results were similar after adjusting for confounders (Table 1), as there was no difference in CRBSI (HR 0.35; *P* = 0.377), thrombosis (HR 0.40; *P* = 0.218), or total events (HR 1.49; *P* = 0.250) between the MC and PICC, while the occlusion was higher in the PICC group (HR 9.81; *P* < 0.001). Vasopressor DOT was independently associated with increased CRBSI (HR 1.16; *P* = 0.006) and total adverse events (HR 1.10; *P* = 0.005), while antibiotic DOT was associated with increased thrombosis (HR 1.07; *P* = 0.017).

**Table 1.** Cox proportional hazard multivariable models for study outcomes

	CRBSI			Thrombosis (DVT or SVT)			Occlusion			Any adverse event		
*Predictors*	*HR*	*CI*	*P*	*HR*	*CI*	*P*	*HR*	*CI*	*P*	*HR*	*CI*	*P*
**Device (PICC vs MC)**	0.35	0.03–3.59	.377	0.40	0.09–1.73	.218	9.81	3.02–31.92	**<.001**	1.49	0.76–2.92	.250
**Age (years)**	1.00	0.95–1.05	.984	1.00	0.97–1.03	.998	0.99	0.96–1.03	.735	1.00	0.98–1.01	.637
**Gender (male vs female)**	2.78	0.47–16.26	.257	0.99	0.41–2.38	.987	1.74	0.55–5.52	.346	1.32	0.71–2.48	.380
**Unit (SICU vs MICU)**	5.24	1.13–24.23	**.034**	1.12	0.38–3.31	.842	1.62	0.50–5.29	.421	1.86	0.95–3.66	.071
**Vasopressor DOT**	1.16	1.05–1.29	**.006**	1.00	0.88–1.14	.993	1.03	0.85–1.24	.785	1.10	1.03–1.18	**.005**
**Antibiotic DOT**	1.07	0.99–1.15	.109	1.07	1.01–1.14	**.017**	0.91	0.82–1.02	.094	1.02	0.98–1.06	.337
Observations	445			445			442			442		
R2Nagelkerke	0.313			0.038			0.175			0.054		

Note. CRBSI, catheter-related bloodstream infections; DVT or SVT, deep vein thrombosis or superficial venous thrombosis; HR, hazard ratios; CI, confidence interval; PICC, peripherally inserted central catheters; MC, midline catheters; SICU, Surgical Intensive Care Unit; MICU, Medical Intensive Care Unit; DOT, days of therapy.

## Discussion

Our review suggests no difference between MC and PICC in CRBSI, thrombosis, or total adverse events; however, occlusion was higher in the PICC group. This aligns with previous studies suggesting that MC and PICC carry comparable risk profiles. This supports the idea that the choice of line type for hospitalized patients requiring short-term therapy need not be limited to CVC or PICC, even in patients requiring certain vesicant antibiotics or vasopressors. Strengths of our study included the absence of crossover between patients with MC and PICC, excluding patients with CVC, and controlling for the medication type infused through each line.

Our findings differ from a recent meta-analysis (3) that suggested that MC may be superior to PICC when comparing infection risk. One potential explanation for the difference in results may lie in the vesicant nature of the medications selected. Indeed, vasopressor DOT were independently associated with a small yet significant increase in the risk of CRBSI and total adverse events, while antibiotic DOT were associated with a small risk for thrombosis. Importantly, this association was irrespective of device type, and our study did not have sufficient power to detect between-group differences.

Our study has several limitations. The occlusion of MC may have been under-identified as MC may have been replaced rather than salvaged with alteplase if found nonfunctioning We did not have information on the number of lumens in each device, which made it not possible to assess the role of the number of lumens on device outcomes. Blood cultures were manually reviewed non-blinded, leading to potential information bias. We have included several potential confounders in the multiple regression models (age, ICU type, DOT). Still, it is possible that there are differences in outcomes related to unmeasured confounders (eg, comorbidities). Our study was insufficiently powered to ascertain the true differences between MC and PICC. Finally, the findings are limited to one academic medical center, limiting generalizability.

## Conclusion

Our study suggests no difference between CRBSI, thrombosis, or total adverse events rates in hospitalized patients receiving vesicant medications through either MC or PICC; however, the study was not powered enough to detect differences. Larger studies are needed to assess the safety of administering these medications via MC in hospitalized patients.

## References

[1] Turcotte S , Dubé S , Beauchamp G. Peripherally inserted central venous catheters are not superior to central venous catheters in the acute care of surgical patients on the ward. World J Surg 2006;30:1605–1619. doi:10.1007/s00268-005-0174-y 16865322

[2] Chopra V , O’Horo JC , Rogers MA , Maki DG , Safdar N. The risk of bloodstream infection associated with peripherally inserted central catheters compared with central venous catheters in adults: a systematic review and meta-analysis. Infect Control Hosp Epidemiol 2013;34:908–918. doi:10.1086/671737 23917904

[3] Urtecho M , Torres Roldan VD , Nayfeh T , et al. Comparing complication rates of midline catheter vs peripherally inserted central catheter. A systematic review and meta-analysis. Open Forum Infect Dis 2023;10:ofad024. doi:10.1093/ofid/ofad024 36751645 PMC9898877

[4] Swaminathan L , Flanders S , Horowitz J , Zhang Q , O’Malley M , Chopra V. Safety and outcomes of midline catheters vs peripherally inserted central catheters for patients with short-term indications: a multicenter study. JAMA Intern Med 2022;182:50–58. doi:10.1001/jamainternmed.2021.6844 34842905 PMC8630646

[5] Gorski LA , Hadaway L , Hagle ME , et al. Infusion therapy standards of practice. J Infus Nurs. 2021;44(1S):S1–224. 33394637 10.1097/NAN.0000000000000396

[6] Gorski LA . The 2016 infusion therapy standards of practice. Home Healthc. Now. 2017 ;35(1):10–8. 27922994 10.1097/NHH.0000000000000481

[7] Caparas JV , Hung HS. Vancomycin administration through a novel midline catheter: summary of a 5-year, 1086-patient experience in an urban community hospital. J Assoc Vasc Access 2017;22:38–41.

[8] Prasanna N , Yamane D , Haridasa N , Davison D , Sparks A , Hawkins K. Safety and efficacy of vasopressor administration through midline catheters. J Crit Care 2021;61:1–4. doi:10.1016/j.jcrc.2020.09.024 33049486

[9] Gershengorn HB , Basu T , Horowitz JK , et al. The association of vasopressor administration through a midline catheter with catheter-related complications. Ann Am Thorac Soc 2023;20:1003–1011. doi:10.1513/AnnalsATS.202209-814OC 37166852 PMC12335012

